# Candida Periprosthetic Joint Infection: Is It Curable?

**DOI:** 10.3390/antibiotics10040458

**Published:** 2021-04-17

**Authors:** Laura Escolà-Vergé, Dolors Rodríguez-Pardo, Pablo S. Corona, Carles Pigrau

**Affiliations:** 1 Infectious Diseases Department, Hospital Universitari Vall d’Hebron, Universitat Autònoma de Barcelona, Passeig Vall d’Hebron 119-129, 08035 Barcelona, Spain; dolorodriguez@vhebron.net (D.R.-P.); cpigraus@gmail.com (C.P.); 2 Spanish Network for Research in Infectious Diseases (REIPI RD16/0016/0003), Instituto de Salud Carlos III, 28029 Madrid, Spain; pcorona@vhebron.net; 3 Study Group on Osteoarticular Infections of the Spanish Society of Clinical Microbiology and Infectious Diseases (GEIO-SEIMC), 28003 Madrid, Spain; 4 Septic and Reconstructive Surgery Unit (UCSO), Orthopaedic Surgery Department, Vall d’Hebron University Hospital, Passeig Vall d’Hebron 119-129, 08035 Barcelona, Spain

**Keywords:** *Candida* spp., periprosthetic joint infection, fungus, biofilm, antifungal-loaded cement spacer, two-stage exchange surgery

## Abstract

Candida periprosthetic joint infection (CPJI) is a rare and very difficult to treat infection, and high-quality evidence regarding the best management is scarce. *Candida* spp. adhere to medical devices and grow forming biofilms, which contribute to the persistence and relapse of this infection. Typically, CPJI presents as a chronic infection in a patient with multiple previous surgeries and long courses of antibiotic therapy. In a retrospective series of cases, the surgical approach with higher rates of success consists of a two-stage exchange surgery, but the best antifungal treatment and duration of antifungal treatment are still unclear, and the efficacy of using an antifungal agent-loaded cement spacer is still controversial. Until more evidence is available, focusing on prevention and identifying patients at risk of CPJI seems more than reasonable.

## 1. Introduction

Periprosthetic joint infection (PJI), which occurs in approximately 1–2% of all procedures, is one of the most feared complications after arthroplasty due to its associated comorbidities and the possible need for implant removal [1]. Candida periprosthetic joint infection (CPJI) represents a rare etiology among all PJIs; sometimes it is very difficult to diagnose, and it is especially difficult to treat when the prosthetic material cannot be removed [2]. In addition, we have no clear guidelines regarding the best antifungal management in these cases [3,4,5,6,7], and evidence is based on small retrospective series.

## 2. Epidemiology

There have been a few recent studies analyzing the prevalence of these infections, and most of them are retrospective in nature [8,9,10,11]. A Spanish retrospective multicenter study that analyzed the etiology of PJIs from 2003 to 2012 found that a fungal etiology represented 1.3% of all culture-positive PJIs (*n* = 2288), and *Candida* spp. were responsible for 90% of all fungal infections [9]. A smaller retrospective multicenter study performed in Australia from 2006 to 2008 found that CPJI accounted for 0.7% (1/152) of all culture-positive infections [10], and another study that compared the etiology of PJIs between two referral centers in Europe and in the United States between 2000 and 2011 found that fungal PJIs were responsible for 2.3% of 772 cases and 0.3% of 898 cases, respectively [11].

The species of *Candida* depends on the local epidemiology of the geographical area. In two multicenter studies in Spain [2,9] and one in the United States [12], *C. albicans* was the most frequently isolated fungus (55–65%), followed by *C. parapsilosis* (13–33%). Other species, such as *C. glabrata* and *C. tropicalis,* were more anecdotic (3–7% and 2–4%, respectively). Smaller series have found similar results [13,14], and in a recent review of the literature, *C. albicans* (47.3%) was the most frequent strain isolated, followed by *C. parapsilosis* (22.3%) [15], but epidemiology may still vary among regions.

## 3. Pathogenesis and Risk Factors

Colonization by *Candida* spp. is regarded as the first step for subsequent infection [16], and *Candida* spp. are common commensals of the human skin and gut microbiota in healthy individuals [17,18,19]. Invasive disease, which encompasses both candidemia and deep-seated infections, usually results from an abnormal or increased number of fungi combined with alterations in the cutaneous and mucosal barriers due to weakening of host immunity [16,17], which permits the transition from *Candida* sp. commensalism to opportunism. Three possible routes of CPJI development have been described: (1) the hematogenous route from an infected catheter or a urinary or intraabdominal source; (2) direct inoculation during prosthesis implantation, revision surgery, or even after arthrocentesis, especially in colonized patients; and (3) extension into synovial fluid from contiguous infected tissues.

*Candida* spp. have specific properties allowing them to adhere to surfaces and form biofilms, especially on prosthetic devices, which permits the development of persister cells, facilitating antifungal resistance, and explains treatment failure when the implant is not removed. In vitro experiments have shown that *C. albicans* biofilm formation begins with the adherence of yeast to a substrate and thereafter yeast cells proliferate across the surface and produce filamentous forms, including hyphae and pseudohyphae. As the biofilm matures, an extracellular matrix accumulates, facilitating antifungal resistance, notably to azoles and polyenes, through different mechanisms [20], which may explain the high failure rates in CPJI when the implant is not removed. Finally, non-adherent yeast cells are released from the biofilm into the surrounding medium (the dispersal step). *C. albicans*, the most frequent causative agent of CPJI, has been reported to form larger and more complex biofilms than other *Candida* species [21]. 

All parts of the immune system are involved in the response to this infection. For example, deficiencies in the T-helper 17 lymphocyte cell line impair the mucosal immune response to *Candida* spp. and facilitate Candida infections. Neutrophil dysfunction or leukopenia also predisposes patients to suffer invasive candidiasis, and complement or immunoglobulin deficiency or alteration is associated with complicated disease as well [17]. The regulatory pathways and mechanisms that govern Candida biofilm development are very complex [20]; gene expression of *C. albicans* is regulated by both a continuous host–pathogen interplay and by distinct genetic mechanisms [19], but this is not the scope of this review.

However, there are other factors that are not only easier to identify than alterations in host immunity but also probably more prevalent in patients with CPJIs and may play a major role in the pathogenesis of invasive candidiasis. The most reported factors are as follows [17]: (1) the long-term or repeated use of broad-spectrum antibiotics, especially in the previous 3 months, which depletes commensal gut bacteria, enabling *Candida* sp. overgrowth. Many antibiotics are known to promote fungal growth and pathogenicity because they disrupt the microbiota and eliminate anaerobic bacteria in the gut which could have otherwise inhibited the fungi, and studies show that the introduction of small amounts of *C. albicans* to mice after antibiotic treatment caused significant changes in the gut microbiota, which may persist in the long term [22]. (2) Breach of the cutaneous and gastrointestinal barriers by chemotherapy, surgery, gastrointestinal perforation, or instrumentation, such as central venous catheters, which may facilitate *Candida* sp. translocation into the bloodstream. (3) Immunosuppression secondary to malignant diseases, immunodeficiencies, or immunosuppressive therapy. Other risk factors reported in patients with CPJIs have been older age [18], diabetes, rheumatoid arthritis, malnutrition, and tuberculosis, which probably also reflect alterations in host immunity [2,12,13,14,23]. Other series have also identified that multiple previous surgeries at the site of the CPJI are also a risk factor [2,13,23,24]. A recent retrospective case–control study that compared fungal PJIs with bacterial PJIs found that recent antibiotic consumption (OR: 3.4; 95% CI: 1.2–9.3) and prolonged wound drainage (OR: 7.3; 95% CI: 2.02–26.95) were significantly associated with CPJI [13]. In our experience, patients treated with long courses of linezolid for multidrug-resistant chronic bacterial PJIs tend to present mucocutaneous candidiasis, and their colonization may persist for an unknown duration, which could also be another risk factor for hip CPJI.

Although it has not been deeply studied, considering the pathogenesis of the disease, previous *Candida* spp. colonization in the urine or Candida intertrigo may also be risk factors in patients undergoing hip arthroplasty [2,13]. In a multicenter retrospective study of patients with CPJIs, we found 14% of patients with Candida intertrigo and 9% of patients with a previous urinary tract infection (three with positive blood cultures) caused by the same *Candida* spp. before the diagnosis of CPJI [2]. 

## 4. Clinical Manifestations and Diagnosis

CPJIs are usually chronic infections characterized by pain, swelling, and sinus tracts. Implant loosening may be observed on radiography in nearly 50% of cases, as previously reported in some studies [2,25]. In fact, the median duration from the index surgery and the diagnosis of CPJI averaged 17–25 months [12,13]. Blood tests could show no leukocytosis, and the C-reactive protein (CRP) level and erythrocyte sedimentation rate are usually normal or mildly elevated [2,12]. The same recently published study comparing patients with CPJIs with those with bacterial PJIs showed that patients with CPJIs had lower median CRP values (2.95 mg/dL vs. 5.99 mg/dL) and lower synovial fluid leukocyte levels (13,953 cells/mm^3^ vs. 33,198 cell/mm^3^) [13].

**Figure 1:**
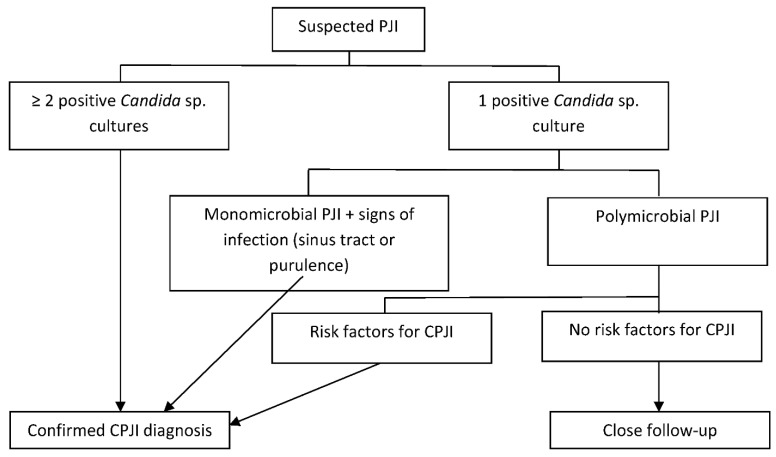
Diagnosis of Candida periprosthetic joint infection.

The criteria to diagnose CPJI are not well established, and the same criteria used in diagnosing bacterial PJIs may not be reliable in some cases. The Infectious Diseases Society of America (IDSA) guidelines [3], a previous International Consensus on PJIs [6], and a recent European Bone and Joint Infection Society (EBJIS) consensus [26] consider two or more intraoperative cultures or the combination of preoperative aspiration and intraoperative cultures yielding the same organism definitive evidence of a PJI [3]. However, when reviewing published series of CPJI cases, the microbiological criteria changed from one study to another. Some authors consider that one positive preoperative aspiration culture and/or a positive intraoperative culture is sufficient [9], while others require two positive cultures [2,13] or one positive culture with additional criteria for PJIs [2,24]. In our opinion, when *Candida* spp. are found in only one intraoperative culture, the case should be evaluated carefully, and treating the Candida etiology should be considered, especially in patients with other risk factors for CPJI such as previous antibiotic therapy or multiple previous surgeries (Figure 1). In fact, even if another microorganism is isolated in two or more cultures, polymicrobial infection is not infrequent, particularly in the hip location, being found in 16% to 26% of cases, depending on the series [2,12], and this should not be a criterion for discarding the value of one positive culture for *Candida* spp.

## 5. Medical and Surgical Treatment

International guidelines on candidiasis and PJIs [5,7] recommend, with limited evidence, the combination of prosthesis removal and reimplantation in two stages. They recommend a prolonged period of antifungal therapy for at least 12 weeks after resection arthroplasty and at least 6 weeks after prosthesis implantation, without specifying the best antifungal option [5]. They state that the use of antifungal agent-loaded cement spacers is controversial.

The fact that *Candida* spp. grow and form biofilms on medical devices makes these microorganisms highly resistant to antifungal agents and the host immune system [27,28,29,30]. Therefore, the best surgical approach is to remove the prosthetic material to avoid the problem of antifungals penetrating and acting within the biofilm. In this sense, a two-stage exchange arthroplasty strategy is probably the best option when feasible to eradicate the infection and to preserve joint function [15], with variable success rates from 14% to almost 100% depending on the series and on the definition of success [2,12,14,23,24,25,31,32,33,34,35]. In patients with reduced mobility, particularly old patients with multiple previous surgeries in the same location, a resection arthroplasty may be the best alternative. There is less evidence of success with a one-stage exchange arthroplasty strategy, which has been reported in only a few cases [15,36,37]. In a recent review of the literature of 76 episodes of CPJI, one-stage exchange arthroplasty was performed only in three patients with a favorable outcome [15], but in another series of 11 CPJI episodes, it was performed in four with success in two [14]. However, due to the publication bias, the small amount of experience and the difficulty of curing this type of infection, with a high rate of relapses, in our opinion, this procedure should be used only in very selected cases. Irrigation and debridement with prosthesis retention usually fails to cure the infection (cure rates from 0% to 20%), especially in cases of chronic infection [2,12,23,32,35]. Table 1 summarizes the type of treatment, the duration of follow-up and the outcome of the larger case series (number of patients ≥ 10) of CPJI.

**Figure 2:**
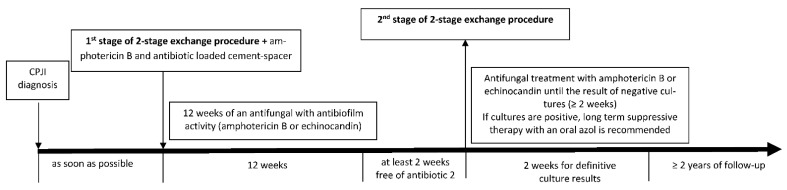
Our proposal for optimal treatment of Candida periprosthetic joint infection.

Fluconazole is active against most CPJI isolates, and it shows good penetration into synovial fluid and less toxicity than amphotericin B, but its activity against *Candida* sp. biofilms is limited. However, the antifungals that have demonstrated better activity against biofilms are echinocandins and liposomal formulations of amphotericin B [27,28,29,38]. In the absence of clear recommendations for systemic antifungal treatment, the most frequently used antifungals have been fluconazole followed by amphotericin B in older series and [15] by echinocandins in recent series [2], with different outcomes, especially in relation to the type of surgical approach (Table 1). However, due to the rarity of this infection, there will probably not be randomized clinical trials regarding the best antifungal treatment. In our retrospective multicenter study, we found better results when amphotericin B or echinocandins rather than fluconazole were combined with implant removal [2], with remission rates higher than 80% vs. 62%, similar to values reported in previous studies [32,39]. Therefore, we would recommend the use of an antifungal with antibiofilm activity, amphotericin B or an echinocandin, after resection arthroplasty and after prosthesis implantation, following our proposed diagram of treatment in Figure 2. 

On the other hand, few studies have evaluated the efficacy of using an antifungal agent-loaded cement spacer in staged exchange arthroplasty for CPJI, so the indication to use it remains controversial. Moreover, there is no consensus on which antifungal agent should be used and at what dose to achieve the optimal balance between cement stability and drug elution. There have been some cases in which amphotericin B deoxycholate or an azole (mainly fluconazole or voriconazole) was mixed with the cement in the spacer [2,15,35,40,41,42], with different outcomes. In our clinical practice, amphotericin B (200 mg of amphotericin B deoxycholate for every 40 g of bone cement) is often used because of its broad antifungal spectrum and antibiofilm activity, its heat stability, and its availability in powder form. However, amphotericin B has been proven to behave differently than water-soluble antibacterial agents [43,44], and it is not clear whether the local dose is sufficiently high to elute from cement spacers [27,39,42,43,44] or whether it is toxic to osteoblasts [45]. An in vitro study found that the elution of 800 mg of liposomal amphotericin B was higher than that of the same dose of deoxycholate amphotericin B when mixed with acrylic bone cement, although it was associated with a loss of compressive strength [46]. In addition, some authors and ourselves have concerns about using only antifungal agents in cement spacers, and we prefer to combine amphotericin B with vancomycin plus gentamycin to avoid bacterial superinfections [15]. Until more evidence is available, we believe that using antifungal agent-loaded cement spacers (preferably with amphotericin B and combined with antibacterial agents) in staged exchange arthroplasty seems reasonable to avoid relapses secondary to fungi that may remain adhered to the bone and cement spacer.

Another unsolved issue is the duration of antifungal treatment. Although short antifungal courses (6 weeks) were successful in a small series when using a staged exchange procedure [33], the median duration of antifungal treatment in larger studies was 3 months [2,12,25,34], consistent with IDSA guideline recommendations [5]. In our opinion, at least 3 months of antifungal treatment are necessary, especially with a drug with antibiofilm activity (an echinocandin or amphotericin B) and combined with implant removal whenever possible, preferably in the form of a two-stage exchange procedure to maintain joint functionality (Figure 2). In patients with high surgical risk for whom prostheses cannot be removed, suppressive therapy with azoles may be an alternative treatment to maintain joint functionality [2].

## 6. Prognosis and Prevention

The prognosis of patients with CPJIs varies depending on the medical and surgical approach. Often, aggressive surgical treatment is dismissed due to the patient’s comorbidities, and resection arthroplasty or amputation is performed, resulting in poor patient functionality, but at least curing the infection. On the other hand, even if performing the best strategy (a two-stage exchange), some patients may persist with the infection or relapse. A recent study found that the main risk factors for two-stage exchange failure are hemodialysis, obesity, multiple previous procedures, diabetes, corticosteroid therapy, hypoalbuminemia, immunosuppression, rheumatological diseases, coagulation disorders, and infection due to multidrug-resistant bacteria or fungal species [47]. Therefore, if some of these risk factors coexist in a patient with CPJI, a resection arthroplasty, agreed with the patient, may be the best alternative to cure the infection even if it implies loosing functionality. Unfortunately, we have no score of risk that helps us in making the best decision. In addition, due to the formation of biofilms by Candida spp., CPJIs, when treated, may take several months or even years to relapse. Patient follow-up varies among some studies, and this makes it difficult to establish when CPJI can be considered cured. In our personal experience, due to the chronic nature of CPJI, follow-up periods shorter than 2 years may not be able to detect some relapses.

As histories of previous antibiotic therapy or surgery are not modifiable, we believe that searching for and treating Candida intertrigo in patients with risk factors for CPJI would be a reasonable, cost-effective measure [2,13]. Therefore, although there is no strong evidence to support this hypothesis, we believe that patients with previous Candida infection or clinical Candida colonization may benefit from the addition of fluconazole to standard prophylaxis before hip arthroplasty. Another more debatable measure would be including fluconazole in surgical prophylaxis for patients with an advanced age, diabetes, a long course of antibiotic therapy in the previous months (especially if it was with linezolid) and multiple previous orthopedic surgeries. As these factors may be difficult to evaluate retrospectively, prospective multicenter studies are needed.

Given the poor prognosis of this type of infection, until more evidence is available regarding the best antifungal treatment, the duration of treatment, and the efficacy of using antifungal agent-loaded cement spacers, focusing on CPJI prevention remains essential. 

## Figures and Tables

**Table 1:** Treatment, follow-up, and outcome of the largest series (number of patients ≥ 10) of Candida periprosthetic joint infection.

Study (Year)	Number of Patients (*n*)	Surgical Treatment (*n*)	Antifungal Treatment (*n*)	Antifungal-Loaded Cement Spacers (*n*)	Follow-Up	Outcome (*n*)
Saconi et al. (2020) [14]	11	Resection arthroplasty 6	Fluconazol 6	No	Range 5.6–74 months	Remission 5 Lost to follow-up 1
		One-stage exchange 4	Fluconazol 2 Itraconazol 1 Micafungin 1	No	Range 2.1–84 months	Remission 2 Failure 1 Lost to follow-up 1
		Two-stage exchange 1	Voriconazol, itraconazol 1	No	48 months	Remission 1
Escolà-Vergé et al. (2018) [2]	35	Prosthesis removal 20	Azoles 13 Antibiofilm-containing regimen * 6 No antifungal 1	Amphotericin B 3	24 months	Remission 13 Failure 7
		Debridement with prosthesis retention 15	Azoles 10 Antibiofilm-containing regimen * 5	No	24 months	Remission 4 Failure 11
Brown et al. (2018) [24]	25	Two-stage exchange 11	Fluconazol 25	Amphotericin B 10	Not reported	Remission 5 Failure 6
		Debridement with prosthesis retention 5		No		Failure 5
		Resection arthroplasty 5		No		Remission 2 Failure 3
		Prosthesis retention and suppressive therapy 3		No		Remission 3
		One-stage exchange 1		No		Remission 1
Gao et al. (2018) [35]	14	Two-stage exchange 14	Fluconazol 11 Caspofungin, fluconazol 1 Vorinconazol 1 Amphotericin B, caspofungin, fluconazol, voriconazol 1	Amphotericin B 3 Voriconazol 2 Fluconazol 2	Mean 65.1 months Range 25–129 months	Remission 10 Failure 4
Ueng et al. (2013) [31]	16	Two-stage exchange 9	Fluconazol 9	Amphotericin B 5	Mean 41 months Range 28–90 months	Remission 8 Failure 1
		Resection arthroplasty 7	Fluconazol 7	Amphotericin B 1		Remission 4 Failure 3
Hwang et al. (2012) [32]	28	Two-stage exchange 24	Amphotericin B 21 Amphotericin B, fluconazol 3 Fluconazol 4	No	Mean 4.3 years Range 2.6–6.1 years	Remission 22 Failure 2
		Debridement with prosthesis retention 4		No		Failure 4
García-Oltra et al. (2011) [23]	10	Two-stage exchange 7	Fluconazol 5 Anidulafungin, fluconazol 1 Caspofungin, fluconazol 1	No	Mean 31 months Range 2–67 months	Failure 7
		Debridement with prosthesis retention 3	Fluconazol 3	No		Failure 3
Azzam et al. (2009) [12]	31	Two-stage exchange 19	Fluconazol 23 Caspofungin, fluconazol 3 Amphotericin B 5	Amphotericin B 5	Mean 45 months Range from 24 months to 11 years	Remission 9 Failure 10
		Resection arthroplasty 10 (5 with previous debridement failure)				Remission 6 Failure 4
		Debridement with prosthesis retention 7		No		Failure 7

* Antibiofilm-containing regimen: amphotericin B or an echinocandin.

## Abbreviations

PJI	periprosthetic joint infection
CPJI	Candida periprosthetic joint infection
